# Why Do They Stay? Intention to Stay among Registered Nurses in Nursing Homes

**DOI:** 10.3390/ijerph17228485

**Published:** 2020-11-16

**Authors:** Ji Yeon Lee, Juh Hyun Shin

**Affiliations:** College of Nursing, Ewha Womans University, Seoul 03760, Korea; juhshin@ewha.ac.kr

**Keywords:** nursing home, intention to stay, Korea, multilevel regression analysis

## Abstract

Purpose: The purpose of this study was to examine the factors associated with nursing home (NH) registered nurses’ (RNs’) intention to stay in their workplace. Methods: A cross-sectional questionnaire survey was used in this study. Organizational NH data were acquired from the administrators of 56 NHs. Individual RN data were acquired from 189 RNs in 56 NHs across Korea. The questionnaire assessed RNs’ intention to stay in their workplace as well as potential associated factors, including individual and organizational factors. Multilevel regression analysis was used to determine which factors explain RNs’ intention to stay in their workplace. Findings: NH RNs’ intention to stay was positively associated with RNs’ years of experience in NHs, career promotion opportunities, and perceptions of NH resident safety culture. At the organizational level, no factors were found to significantly relate to the intention to stay of NH RNs. Conclusions: Although this study found that organizational factors have no statistically significant relationship with RNs’ intention to stay, organizational support must precede changes in individual factors that have significant relationships. Clinical Relevance: Organizational (NH) and individual (RN) efforts must be made to enhance RNs’ intention to stay because individual factors can change after implementing efforts such as providing educational programs, promotional opportunities, and forming a positive resident safety culture at an organizational level.

## 1. Introduction

The global population is aging at a faster rate than in the past, and these demographic changes will affect almost every aspect of society. Globally, there are already more than 1 billion people aged 60 or older [1]. Korea’s population is rapidly aging. Korea became an aged society in 2017 (>14% of the total population aged 65 years and older) in only 17 years, a rate that is unprecedentedly fast. The long-term-care social insurance, which was introduced in Korea in 2008, covers care for Korea’s elderly [2]. As a result of this system, the number of nursing homes (NHs) increased more than 2.5 times from only 1332 in 2008 to 3389 in 2019, and during the same period, the number of residents increased more than 2.5 times from 66,715 to 163,484, and this increase is expected to continue [2]. Securing an appropriate level of registered nurses (RNs) in NHs is increasingly important to accommodate the expected increase in the elderly population. This is because RNs are a key force that affects the quality of care for residents in NHs [3]. RNs provide clinical care, care management, and planning for evidence-based practice based on timely assessments of residents’ health conditions [3]. However, the shortage and low retention rate of RNs in NHs is a concern for healthcare professionals worldwide [4,5]. This retention issue affects negative RN outcomes such as burnout, dissatisfaction, and intention to leave [6]. This leads to a low quality of care, which results in increased falls, pressure ulcers, contractures, catheter use, restraint use, acute-care-setting admissions, and a higher mortality rate [4,5,6]. Moreover, organizational costs are increasing due to low quality of care, recruitment needs, and productivity loss [7].

Thus, organizations must focus on retaining RNs capable of planning and caring for this aging and vulnerable population [8,9]. The number of RNs in Korean NHs increased only 1.2 times from 1260 in 2008 to 1472 in 2019 [2]. In addition, NH RN tenure is only 2.2 years, and only 46.5% of NH RNs intend to stay in their current workplace [10,11]. Therefore, it is necessary to identify and strengthen NH RNs’ intention to stay and the factors affecting their retention. This focus can increase an organization’s effectiveness by allowing competent RNs to remove causes and retain their position before actually leaving the organization [12].

The staffing criteria suggested by Korean law (noin-jangki-yoyang-bohum) [2] call for one RN or certified nursing assistant (CNA) per 25 residents. Many administrators replace RNs with CNAs to reduce labor costs and because RNs avoid working in NHs (a total of 1472 RNs and 7806 CNAs currently work in Korean NHs). As a result, the number of residents in the charge of each RN is approximately 100, and this figure is significantly higher than that seen in other countries [2]. RNs also manage and supervise CNAs and care workers (CWs), in addition to nursing work, and perform various administrative and managerial roles in NHs [10,13]. In addition, most RNs do not work in NHs at night because NHs do not have mandatory 24-h RN placement. Most RNs are called in and expected to take emergency measures when a resident safety problem occurs at an NH [14]. NHs are prone to safety accidents due to most residents’ decreased cognitive and physical function [15]. RNs have difficulty performing their nursing duties due to the lack of safety culture; therefore, it is necessary to create a resident safety culture to cope with safety concerns [11,16]. Moreover, job satisfaction and job esteem are low due to low wages and lack of career growth opportunities [17].

Job satisfaction is expected to reduce NH RNs’ intention to stay due to the negative resident safety culture, low staffing, and lack of career growth opportunities, but there is a lack of official discussion or examination surrounding Korean NH RNs’ intention to stay in their workplace [18]. Moreover, few studies examine the factors that affect RNs’ intention to stay in NHs worldwide [19]. Examples of individual factors include years of NH experience, wage, career growth opportunities, and job satisfaction. Organizational factors include RN retention rate, leadership, the welfare system, work flexibility, and job training [20,21,22].

In this study, we comprehensively addressed the organizational and individual factors that may affect NH RNs’ intention to stay because RNs share organizational characteristics, which also affect them. We examined the comprehensive scope of organizational and individual factors to identify factors related to NH RNs’ intention to stay using multilevel regression analysis, a method known to enhance the accuracy of data analysis when analyzing organizational and individual factor data [23,24]. We hypothesized that the individual factors (RNs’ years of experience in NHs, monthly income, career promotion opportunities, job esteem, perceptions of the level of RN staffing adequacy in their workplace, and perceptions of NH resident safety culture (individual level)) and organizational factors (ownership form, number of beds, resident–RN ratio, skill mix, RN retention rate, RNs’ night-shift operating methods, regular job training, and NH resident safety culture (organizational level)) would be associated with the increased intention of RNs to stay in NHs.

## 2. Methods

### 2.1. Design of the Study

We used a multilevel cross-sectional design with a questionnaire survey of NH RNs. Explanatory variables included two levels (the organizational (NH) level and the individual (RN) level).

### 2.2. Sampling and Participants

**Figure 1:**
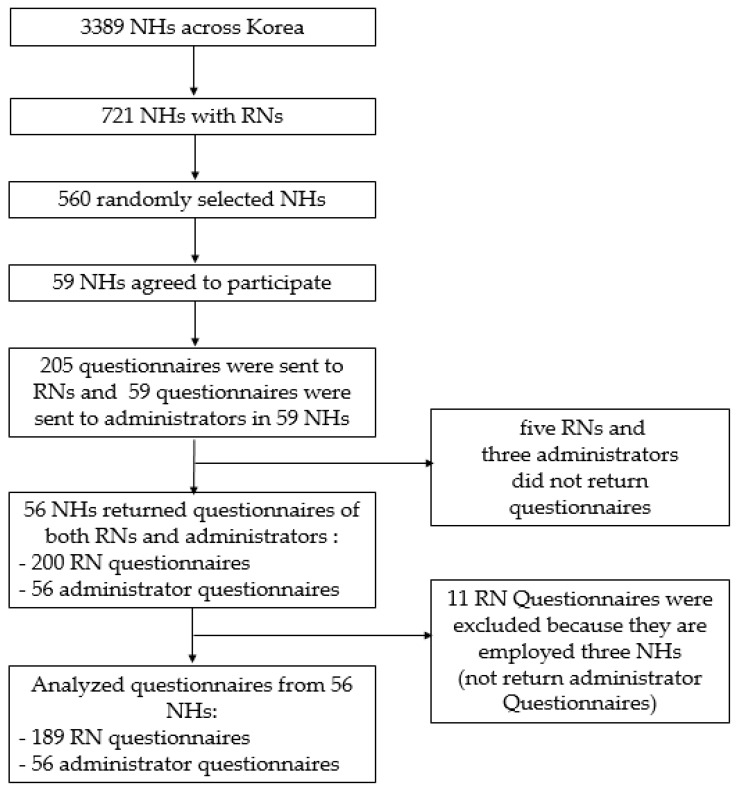
Sampling and participant recruitment.

Data were collected from August 2019 to November 2019. We used a proportional stratified sampling method according to an area of distribution of NHs across Korea to gain representation. The method divided Korea into subgroups aligned with the 17 administrative districts. A total of 3389 NHs exist in Korea, and only 721 of them (21%) deploy RNs. A list of NHs (their name, administrative district, and phone number) in which nurses were deployed was obtained online (www.longtermcare.or.kr). First, we randomly selected a total of 560 NHs that deploy RNs (10–140 NHs in each district) using a table of random numbers from the Korean National Insurance Corporation’s NH list. Second, we called the administrators of those NHs to explain the study’s aim and to ask whether they wished to participate. Administrators of 59 NHs agreed to participate in this study. Third, we visited NHs in person or mailed a cover letter explaining the study’s purpose and methods (for administrators and RNs), organizational factor questionnaires (for administrators), individual factor questionnaires (for RNs), and consent forms (for administrators and RNs) to all administrators and RNs in the NHs that agreed to participate in the study (totaling 59 administrators and 205 RNs; see Figure 1). We asked participants to seal their completed questionnaires in individual envelopes with double-sided tape to promote honest answers and ensure confidentiality. We collected the questionnaires upon completion. Fourth, 56 administrators and 200 RNs responded to the questionnaire and consent form. We excluded 11 RN questionnaires from respondents employed by three NHs for which administrator questionnaires were not returned. Finally, 56 organizational factor questionnaires and 189 individual factor questionnaires were used for analysis.

### 2.3. Sample Size

The sample size in this study was set in consideration of the study method, data collection, and data analysis. There is no clear indication of how large sample sizes must be to produce the appropriate power for multilayer regression analysis in this study [25]. The recommended appropriate level of sampling is 30 (organizational level) [25]. However, this may vary depending on the nature of the study design, and increasing the number of targets at the organizational level rather than increasing the number of targets at the individual level is recommended if data are difficult to obtain [25]. The subject of this study at the individual level is elderly care facility nurses. There are 1472 nurses at senior care centers across the country, and access to this sample was further limited by the number of NHs that allowed RNs to participate in the research. Considering the possibility of collecting and analyzing data, the number of subjects at the organization (senior care facility) level was selected as 56, and 189 nurses working at the elderly care facility were selected for the individual level.

### 2.4. Variables and Measures

The instruments used in this study are all instruments which reported reliability and validity in the preceding study. A group of six experts (two nursing professors and four NH administrators) validated the content validity of all the measures in this study because the measures were developed for acute-care-setting RNs, except for NH resident safety culture. A 5-point Likert scale was established for each item and all items scored 0.8 or higher. Only the term “acute care setting” was changed to “NH” among the items and all the items were used as they were.

**General characteristics.** We collected individual RN characteristics data regarding sex, age, education level, marital status, number of dependent families, years of nursing experience, years of experience in the current NH, position, and employment status from RNs.

### 2.5. Independent Variable

**Individual RN factors.** We included information about RNs’ years of experience in NHs, monthly income, career promotion opportunities, job esteem, perceptions of the level of RN staffing adequacy in their workplace, and perceptions of NH resident safety culture (individual level) in this study. Career promotion opportunity means professional development and workplace learning opportunities, which were assessed by the Questionnaires on the Experience and Evaluation of Work 2.0 (QEEW 2.0) [26]. This scale consists of 6 items on a 5-point scale ranging from 0 (very unlikely) to 4 (very likely). Higher scores indicate greater career promotion opportunity. The Cronbach’s alpha coefficient was 0.87. Job esteem means one’s perception of the level of respect and authority for his or her job. This was assessed by the Job-Esteem Scale for Hospital Nurses (JES-HN) [27]. This scale consists of 28 items and contains 6 subscales, including vocational self-awareness (7 items), vocational competence confidence (5 items), role and expertise of care (4 items), social trust and respect (4 items), respect and recognition of the organization (4 items), and vocational authority and future value (4 items). Each item was rated on a 5-point scale ranging from 1 (very unlikely) to 5 (very likely). Higher scores indicate greater job esteem. The Cronbach’s alpha coefficient was 0.94 for the whole scale and 0.90, 0.88, 0.82, 0.83, 0.87, and 0.80 for each subscale, respectively. Perceptions of the adequacy of the level of nurse staffing in their workplace were assessed by choosing from the following responses: very adequate, adequate, not adequate, or very not adequate. RNs’ perceptions of NH patient safety culture (individual level) refers to how RNs perceive the NH manager’s leadership, nursing staff’s working attitude, and organizational system and patient safety in the NH [28]. This was assessed by the Korean Patient Safety Culture Scale for Nursing Homes [28]. This scale comprises 27 items and contains 4 subscales, including leadership (9 items), organizational system (6 items), working attitude (7 items), and management practice (5 items). Each item was rated on a 5-point scale ranging from 1 (very unlikely) to 5 (very likely). Higher scores indicate a more positive perception of the patient safety culture of the current NH. The Cronbach’s alpha coefficient was 0.95 for the whole scale and 0.93, 0.88, 0.87, and 0.86 for each subscale, respectively. 

**Organizational factors.** We included the ownership form, number of beds, resident–RN ratio, skill mix, RN retention rate, RNs’ night-shift operating methods, regular job training, and NH resident safety culture (organizational level) information as the organizational factors. Skill mix is defined as the proportion of RNs to nursing staff (RN, CNA, and CW). We collected information about the number of RNs, CNAs, and CWs in each NH and calculated skill mix. The Nursing Facility Staff Survey calculated the RN retention rate as the percentage of RNs employed for more than 1 year [29]. We chose the RNs’ night-shift operating methods, such as “operating night shift fixed RN”, “operating rotating shift RN”, “operating without RN at night”, and “calling RN in case of emergency.” We measured regular job training through a dichotomous question (Yes/No). NH administrators completed a survey about the organizational factors, except for NH resident safety culture. We calculated NH resident safety culture (organizational level) as the average of RNs’ NH resident safety culture scores (individual level) at each NH.

**Dependent variable.** We measured the dependent variable—intention to stay—using the RN Retention Index (NRI) [30]. The NRI consisted of 8 items on a 6-point scale ranging from 1 (very unlikely) to 6 (very likely). Higher scores indicate greater intention to stay. The Cronbach’s alpha coefficient was 0.97.

### 2.6. Data Analysis

**Figure 2:**
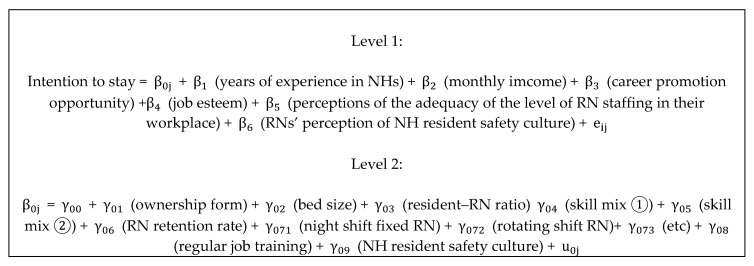
Model specifications of the Level 2 multilevel model regression.

We used multilevel regression analysis to analyze a data structure where RNs (Level 1) were nested within NHs (Level 2). Multilevel regression analysis yields more robust estimates than traditional ordinary least squares when analyzing nested data [23]. Multilevel regression analysis allows simultaneous estimation of relationships in a hierarchical level and relationships between or across hierarchical levels [23]. Model specifications of the Level 2 multilevel model regression are shown in Figure 2.

Level 1 and Level 2 continuous variables (years of experience in NHs, monthly income, career promotion opportunity, job esteem, perceptions of the level of RN staffing adequacy in their workplace, RNs’ perceptions of NH resident safety culture, the number of beds, resident–RN ratio, skill mix, and RN retention rate) in the multilevel model were grand-mean centered to alleviate potential Level 2 estimation problems [31]. The ownership form’s reference variable is profit. Four categories encompassed the RNs’ night-shift operating methods; therefore, three dummy variables (D1: night shift fixed RN; D2: rotating shift RN; D3: etc.) were generated and added to the model using “operating without RN at night” and “calling RN in case of emergency” as reference. The reference variable for regular job training is when regular job training is conducted. MPlus 8.0 [31]. was utilized for multilevel regression analysis.

We estimated 3 models in this study. First, we estimated the null model (i.e., an unconditional model). This model (Model 1) contains no predictors. We examined the variability’s extent of the RNs’ intention to stay across NHs by estimating the null model to account for the nested nature of our data (RNs are nested within NHs). Intraclass correlation (ICC) was 0.721 in this study, indicating that the variability in RNs’ intention to stay at their current workplace is 72.1%. This result justifies the development of a multilevel regression analysis [23]. Second, we estimated an individual-level model (Model 2). This model contains only individual factors. Lastly, we estimated a full model (Model 3). This model contains both individual and organizational factors. The final model provides information on a possible relationship between RNs’ intention to stay and NH characteristics after adjusting for individual-level variables. The Mplus 8.0 software package was utilized for multilevel regression analysis. 

## 3. Results

### 3.1. Characteristics of RNs and NHs

Table 1 shows descriptive statistics for individual (RN)-level variables. All participating RNs were female, and the mean age was 48.52 years. Approximately 51.3% of RNs held an associate degree, 87.4% were married, 70.9% were staff RN, and 84.7% held permanent employment status. Years of total nursing experience, NH experience, and current NH experience were 17.04, 6.19, and 4.75 years, respectively. The monthly income of the majority of RNs ranged between USD 2500 and USD 2990. About half of the RNs (50.8%) perceived the level of RN staffing in their workplace as not adequate. The mean score of career promotion opportunity (professional development and workplace learning opportunity) was 14.44 (range: 0–24), job esteem (one’s perception of the level of respect and authority for his or her job) was 105.81 (range: 28–140), NH resident safety culture (how RNs perceive the NH manager’s leadership, nursing staff’s working attitude, the organizational system, and NH resident safety) was 97.60 (range: 27–135), and intention to stay at one’s workplace was 31.44 (range: 8–48).

Table 2 shows descriptive statics for organizational (NH)-level variables. Most (78.6%) NHs were for-profit. The average number of beds in participating NHs was 101.19. The ratio of RNs to residents was 1:51, ratio of CNAs to residents was 1:59, and ratio of CWs to residents was 1:2. The mean skill mix of nursing staff was 0.51 for RNs/RNs + CNAs and 0.06 for RNs/RNs + CWs. The RN retention rate was 72.2%. The major night-shift operating method is “without RN at night and calling RN in case of emergency” (71.4%). Only 25.0% of NHs offer regular job training for RNs. 

### 3.2. Result of Multilevel Regression Analysis

Table 3 shows the multilevel regression analysis results for intention to stay. We examined the extent to which the combined effects of the Level 1 and Level 2 predictors explain the adjusted variations in the average level of intention to stay. The multilevel regression analysis revealed that NH RNs’ intention to stay is positively associated with RNs’ years of NH experience (β = 0.137, *p* < 0.001), career promotion opportunities (β = 0.338, *p* < 0.001), and perceptions of NH resident safety culture (β = 0.472, *p* < 0.001). In other words, RNs’ increased years of NH experience, career promotion opportunities, and perceptions of NH resident safety culture relate to an increased intention to stay. Model 3 shows that none of the organizational factors are significantly associated with average intention to stay when considering NH cohesion after controlling for individual-level variables.

## 4. Discussion

The World Health Organization (WHO) defined healthy aging as a process of maintaining functional capabilities that enable well-being in old age. The role of RNs in NHs is all the more important for healthy aging [1]. In this context, NH RNs’ intention to stay is an important issue because RNs in NHs contribute to resident health outcomes. However, data on NH RNs’ intention to stay are rare and insufficient for explaining RNs’ intention to stay in their current NH. While there has been some examination on turnover in Korea [32], this study is among the first, to our knowledge, to examine intention to stay among RNs in NHs. This study offers a unique examination of organizational and individual factors and their relationships with NH RNs’ intention to stay. Further, we include rather novel variables that lack investigation in the NH setting, such as RNs’ perceptions of NH resident safety culture, job esteem, and RNs’ night-shift operating methods. This study yielded findings that state RNs’ increased years of experience in NHs, career promotion opportunities, and perceptions of NH resident safety culture relate to an increased intention of RNs to stay in their current workplace.

Years of NH experience significantly contributes to explaining RNs’ intention to stay. Past studies also identified a positive association between intention to stay and NH career [3,19]. The ability to work as a professional and the ability to adapt to work increase as the clinical experience increases and are associated with an increase in job satisfaction and in intention to stay [19,33,34,35]. The factor of years of NH experience is more explanatory in intention to stay than total RN experience [19,33]. The unique role of NH RNs compared to acute-care-setting RNs can explain intention to stay. Emergency situations such as falls, suction, loss of consciousness, and difficulty breathing frequently occur in NHs [15]. Unlike acute care settings, however, physicians are not full-time, requiring RNs to make independent clinical judgments and decisions on emergency situations and administer first-aid to residents. NH RNs must perform administrative and managerial tasks [36], supervise nursing assistant staff [37], and have long-term ties with residents and their families as well, in contrast to the situation faced by acute-care-setting RNs where nursing practice accounts for most RN work. RNs with less experience in NHs will have difficulty performing their duties due to the unique role of NH RNs, even if the RNs have a lot of clinical experience in acute care settings [38,39]. This phenomenon leads to a decrease in intention to stay. Thus, the United States supports improving the job adaptation of newly employed RNs who transition from acute care settings to NHs, or to NHs after college, through the provision of a preceptor and residency program where experienced RNs provide individual orientation and systematic education to RNs who join NHs [20]. The need for appropriate educational programs is all the more necessary in Korea, considering that RNs who quit acute care setting jobs and lose their careers due to childbirth and childcare are often re-employed in long-term care settings after a long recess [40]. Organizational efforts are necessary to develop these programs.

Career promotion opportunity (job training and promotion opportunity) was statistically significantly associated with RNs’ intention to stay, which is consistent with the findings of previous studies [21,22]. Improving professionalism through job training improves job satisfaction, responsibility, and intention to stay [22,41]. RNs at NHs reflected on the need to upgrade their clinical knowledge and skills, given that the resident NH population is increasingly comprised of more medically complex residents [41,42]. Delirium identification and management strategies, dementia nursing, emergency nursing, and abnormal symptom management comprise high-demand job training program content [43,44]. Developing and implementing programs in NHs may be one way to improve RNs’ intention to stay.

Promotional opportunity (another factor in career promotion opportunity) is associated with intention to stay and is one way to manage human resources. This factor increases RNs’ intention to stay by rewarding RNs in a way that promotes organizational commitment [45]. However, few promotional opportunities exist for RNs in NHs due to limited human resources [46,47]. The lack of promotional opportunity is the number one reason for job dissatisfaction among RNs at NHs in Korea. NHs in the United States establish a director of nursing (DON) position and mandate the placement of DONs as well as RNs in all NHs to ensure expertise and RN autonomy [48]. A system must be in place to expand opportunities for RN promotion in Korean NHs.

In this study, we examined whether RNs’ intention to stay is associated with resident safety culture and found that resident safety culture significantly contributes to explaining RNs’ intention to stay. Several studies have investigated the relationship between patient safety culture in acute care settings and intention to stay. Hospital RNs’ positive perceptions of patient safety culture positively relate to intention to stay [49,50]. In the event of a resident safety problem in an organization where RNs’ perceptions of NH resident safety culture is positive, the administrator creates a supportive atmosphere where they check the cause in the organization’s system through open communication without blaming the RN so the RN can continue their job by trusting and engaging with the organization [22]. However, scholars have not yet studied this relationship in NHs. This is the first study to examine the relationship between RNs’ intention to stay and resident safety culture in NHs. NHs do not foster open communication about resident safety issues or mistakes compared to acute care settings [11,51]. In particular, CNAs and CWs (nonmedical Korean NH nursing staff) account for 98.1% of the total nursing staff in Korean NHs and provide direct care to the residents [2]. CNAs and CWs have a low level of understanding of the elderly and provide nonstandardized care built on experience rather than professional knowledge. Thus, the resident safety culture of NHs may be threatened. RNs who must supervise CNAs and CWs have a psychological burden that negatively affects their intention to work [42,52]. Hence, organizational efforts must establish a first-aid procedure and guidelines, acute care setting transfer procedures and guidelines, and an accident reporting system [49], which are key elements in creating a positive resident safety culture. In addition, the organization’s RN leaders must establish and ensure communication and cooperation systems among nursing staff. These efforts will positively affect the RNs’ intention to stay if a positive resident safety culture forms and the RNs perceive it positively.

Unlike the results of the preceding study [6], organizational factors (that is, NH nurse staffing and nurse work environments such as RNs’ night-shift operating methods and NH resident safety culture) were not found to be associated with RNs’ intention to stay. In this context, is important to note that there is no fixed number of residents per RN, which is a result of the chronic RN shortage in NHs across Korea. The staffing criteria suggested by Korean law require 1 RN or CNA per 25 residents; many administrators replace RNs with CNAs to reduce labor costs. Another possible interpretation for this lack of association could be related to the residents’ severity of health problems and the organization not considering the subsequent nursing request. In the United States, according to the residents’ nursing needs, the residents are divided into 66 resource utilization groups (resource utilization groups, version IV, (RUG-IV)) and the nursing staff’s hours per resident day (HPRD) are presented differently for each group [5]. In Japan, nurse staffing standards are set differently depending on the type of facilities for the elderly, considering the nursing needs of the residents. On the other hand, the type of NH and the standard for nurse staffing are unified without considering the degree (Grade 1, those who absolutely need help in their daily lives; Grade 2, those who need much help; Grade 3, those who need help in part; Grade 4, those who need some help; and Grade 5, residents with dementia) to which the residents in Korea need help due to physical and mental dysfunction [18]. The higher the residents’ severity of health problems, the more professional nursing services and nursing hours are required; thus, RNs’ intention to stay decreases due to the burden of work. Therefore, the nurse staffing standard in Korean NHs should also be determined in consideration of the residents’ severity of health problems. Future studies must also consider the residents’ severity of health problems and identify factors that affect NH RNs’ intention to stay. 

This present study provides important insight into administrators and policy decision-makers to support NH RN-focused approaches to increase RNs’ intention to stay. Although this study found that organizational factors have no statistically significant relationship to RNs’ intention to stay, organizational support must precede changes in individual factors that have significant relationships. Administrators must take a proactive approach, such as providing preceptor programs, residency programs, educational programs, and promotional opportunities, and they must form a positive NH resident safety culture. Such organizational support will increase the NH experience of RNs, raise awareness of career growth opportunities, and influence positive awareness of resident safety culture, which are variables identified in this study as individual factors affecting RNs’ intention to stay.

### 4.1. Implications for Clinical Nursing Practice

This study shows that important factors related to NH RNs’ intention to stay are individual factors, but changes can only occur if individual and organizational efforts are made together. For instance, it suggests the need to provide residency programs to improve work adaptation to increase the RNs’ clinical NH experience. Policy support should be supported to enable NHs to provide such programs. Future studies must identify residency program requirements of NH RNs. RNs’ promotion opportunities are limited at NHs due to limitations in human resources and lack of awareness of their expertise in the role of nurses [47]. Especially considering the fact that the average number of NHs in Korea is 48.24 and the standard for nurse staffing at NHs is 1 RN or CNA per 25 residents, it is difficult not only to promote their positions but also to promote them through rotational work between departments. Lack of promotional opportunities lowers RNs’ intention to stay, so organizational support for establishing a promotional system should be prioritized. This will allow RNs to recognize promotion opportunities and improve their intention to stay in NHs. It is also necessary to make organizational efforts to create a positive resident safety culture and to ensure that individuals can positively recognize the resident safety culture in NHs.

### 4.2. Limitations

The present study has a limitation. Although this study used a proportional stratified sampling method according to an area of distribution of NHs across Korea to gain representation, generalizations from this study must be made with caution due to a limitation. The sample of this study (NHs) did not reflect all NHs in Korea. Compared to 2.04 deployed RNs per NH in Korea, 3.38 RNs per NH participated in the study, indicating that the average NH considered in this study has a relatively high distribution of RNs. In addition, while 96.9% of Korean NHs are for-profit NHs [2], only 78.6% of those participating in this study were for-profit NHs, indicating that nonprofit NHs were included at a high rate in this study. As a result, this potential selection bias may have led to the underrepresentation of NHs in Korea. More research throughout Korea is necessary to confirm NH RNs’ intention to stay.

## 5. Conclusions

This study examined which individual and organizational factors relate to NH RNs’ intention to stay in Korea. Individual RN-related factors, namely RNs’ years of NH experience, career promotion opportunities, and perceptions of NH resident safety culture are significant factors that are associated with intention to stay; however, it has not been confirmed that organizational factors are associated with intention to stay. It is necessary to provide appropriate support from the organization level, such as by creating residency programs to improve work adaptation to increase the RNs’ clinical NH experience, establishing a promotional system, and forming a positive resident safety culture. Political support will provide such things on an organizational level, and these efforts will enable individual-level changes.

## Figures and Tables

**Table 1:** Descriptive statistics for individual (resident nurse (RN))-level variables and intention to stay.

Variable	Label (Range)	n	%	M ± SD
Sex	Female	189	100	
Age				48.52 ± 8.14
Education	Associate degree; 2- or 3-year college	97	51.3	
	Bachelor’s degree; university	77	40.7	
	Master’s degree or higher	15	8.0	
Marital status	Unmarried	12	6.3	
	Married	165	87.4	
	Divorce	8	4.2	
	Bereavement	4	2.1	
The number of dependent families				1.65 ± 1.26
Position	Staff RN	134	70.9	
	≤Head RN	55	29.1	
Employment status	Full time	160	84.7	
	Part time	29	15.3	
Years of nursing experience				17.04 ± 7.98
Years of experience in any NHs				6.19 ± 3.58
Years of experience current NH				4.75 ± 3.61
Monthly income (USD)	<2000	10	5.3	
	2000–2490	64	33.8	
	2500–2990	74	39.2	
	3000–3490	35	18.5	
	3500–3990	4	2.1	
	≥4000	2	1.1	
Career promotion opportunity	Total (0–24)			14.44 ± 4.11
Job esteem	Total (28–140)			105.81 ± 15.89
	Vocational self-awareness (7–35)			27.75 ± 4.27
	Vocational competence confidence (5–25)			19.17 ± 3.18
	Role and expertise of care (4–20)			15.67 ± 2.85
	Social trust and respect (4–20)			15.42 ± 2.75
	Respect and recognition of the organization (4–20)			13.56 ± 2.68
	Vocational authority and future value (4–20)			14.25 ± 2.81
RNs’ perceptions of NH resident safety culture	Total (27–135)			97.60 ± 14.15
	Leadership (9–45)			16.24 ± 2.96
	Working attitude (6–30)			22.83 ± 3.60
	Organizational system (7–35)			22.83 ± 3.60
	Management practice (5–25)			16.24 ± 2.96
Perceptions of the adequacy of the level of RN staffing in their workplace	Very adequate	6	3.2	
	Adequate	79	41.8	
	Not adequate	96	50.8	
	Very not adequate	8	4.2	
Intention to stay	Total (8–48)	31.44 ± 9.55		

**Table 2:** Descriptive statistics for organizational (nursing home (NH))-level variables.

Variable	Label (Range)	*n*	%	M ± SD
Ownership	Profit	44	78.6	
	Nonprofit	12	21.4	
Bed size				101.19 ± 58.62
Ratio of staff to residents	RN–residents	1:51		
	CNA–residents	1:59		
	CW–residents	1:2		
Skill mix ① The number of RNs/(the number of RNs + CNAs)				0.51 ± 0.27
Skill mix ② The number of RNs/(the number of RNs + CWs)				0.06 ± 0.03
RN retention rate (%)				72.18 ± 31.04
RNs’ night-shift operating methods	Without RN at night and calling RN in case of emergency	40	71.4	
	Night shift fixed RN	2	3.6	
	Rotating shift RN	11	19.6	
	Etc.^+^	3	5.4	
NH resident safety culture				88.74 ± 15.69
Regular job training	Yes	14	25.0	
	No	42	75.0	
Regular job training hours/year (if yes)				1.86 ± 4.08

Note: S = standard deviation; RN = registered nurse; CAN = certified nursing assistant; CW = care worker; NH = nursing home; ^+^ without RN and CNA at night and calling CNA in case of emergency.

**Table 3:** Multilevel regression analysis for RNs’ intention to stay in NHs.

Variable	Label	Null Model		Model 2		Model 3	
**Fixed effects**		**β**	***p***	**β**	***p***	**β**	***p***
	Intercept ($\gamma_{00}$)	2.866	<0.001 ***	19.753	<0.001 ***	20.907	<0.001 ***
**Level 1: Individual Factors**							
Years of experience in NH				0.137	<0.001 ***	0.130	<0.001 ***
Monthly income				−0.019	0.614	−0.010	0.777
Career promotion opportunity				0.388	<0.001 ***	0.355	<0.001 ***
Job esteem				0.064	0.132	0.056	0.179
RNs’ perceptions of NH resident safety culture				0.472	<0.001 ***	0.510	<0.001 ***
Perception of the adequacy of the level of RN staffing in their workplace				0.008	0.740	0.011	0.605
**Level 2: Organizational Factors**							
Ownership: Profit						−0.153	0.399
Bed size						−0.209	0.267
Ratio of RNs to residents						−0.022	0.947
Skill mix ① The number of RNs/ (the number of RNs + CNAs)						0.644	0.185
Skill mix ② The number of RNs/ (the number of RNs + CWs)						0.186	0.555
RN retention rate						0.127	0.573
RNs’ night-shift operating methods	Night shift fixed RN					−0.579	0.060
	Rotating shift RN					−0.008	0.978
	Etc.					0.054	0.668
	Without RN at night and calling RN in case of emergency					reference	reference
Regular job training: Yes						0.029	0.874
NH resident safety culture						−0.454	0.316
**Random effects**		**β**	***p***	**β**	***p***	**β**	***p***
Variance of the individual level ($\sigma^{2}$)		33.003	<0.001 ***	7.859	<0.001 ***	7.727	<0.001 ***
Variance of the organizational level ($\tau^{2}$)		84.733	<0.001 ***	2.497	0.387	0.722	0.481
${R_{1}}^{2}$				0.762		0.766	
${R_{2}}^{2}$						0.991	
ICC		0.721					

*** *p* < 0.001.
